# Experimental and computational study on anti-gastric cancer activity and mechanism of evodiamine derivatives

**DOI:** 10.3389/fphar.2024.1380304

**Published:** 2024-05-07

**Authors:** Jingli Liu, Yingying Xue, Kaidi Bai, Fei Yan, Xu Long, Hui Guo, Hao Yan, Guozheng Huang, Jing Zhou, Yuping Tang

**Affiliations:** ^1^ College of Pharmacy, Shaanxi University of Chinese Medicine, Xianyang, Shaanxi, China; ^2^ Key Laboratory of Shaanxi Administration of Traditional Chinese Medicine for TCM Compatibility, Shaanxi University of Chinese Medicine, Xianyang, Shaanxi, China; ^3^ College of Chemistry and Chemical Engineering, Anhui University of Technology, Ma’anshan, Anhui, China

**Keywords:** evodiamine derivative, anti-gastric cancer activity, MTT, DFT, molecular docking, molecular dynamic simulation

## Abstract

**Introduction:** Human topoisomerase 1 (TOP1) is an important target of various anticancer compounds. The design and discovery of inhibitors targeting TOP1 are of great significance for the development of anticancer drugs. Evodiamine and thieno [2,3-d] pyridine hybrids show potential antitumor activity. Herein, the anti-gastric cancer activities of these hybrids were investigated.

**Methods:** The inhibitory effects of different concentrations of ten evodiamine derivatives on the gastric cancer cell line SGC-7901 were assessed using a methyl thiazolyl tetrazolium assay. Compounds EVO-1 and EVO-6 strongly inhibited gastric cancer cell proliferation, with inhibition rates of 81.17% ± 5.08% and 80.92% ± 2.75%, respectively. To discover the relationship between the structure and activity of these two derivatives, density functional theory was used to investigate their optimized geometries, natural population charges, frontier molecular orbitals, and molecular electrostatic potentials. To clarify their anti-gastric cancer mechanisms, molecular docking, molecular dynamics simulations, and binding free energy calculations were performed against TOP1.

**Results:** The results demonstrated that these compounds could intercalate into the cleaved DNA-binding site to form a TOP1–DNA–ligand ternary complex, and the ligand remained secure at the cleaved DNA-binding site to form a stable ternary complex. As the binding free energy of compound EVO-1 with TOP1 (−38.33 kcal·mol^−1^) was lower than that of compound EVO-6 (−33.25 kcal·mol^−1^), compound EVO-1 could be a more potent anti-gastric cancer agent than compound EVO-6.

**Discussion:** Thus, compound EVO-1 could be a promising anti-gastric cancer drug candidate. This study may facilitate the design and development of novel TOP1 inhibitors.

## 1 Introduction

Gastric cancer (GC) is a common malignant tumor of the digestive system, characterized by high metastasis, mortality, and recurrence rates (Sexton et al., 2020). According to the cancer statistics data of 2020 (Sung et al., 2021), GC ranks fifth (5.6%) among commonly diagnosed cancers and fourth (7.7%) among cancer deaths worldwide. Although the incidence rate of GC has gradually declined in recent years, further treatment development will still have an important impact on global health (Thrift and El-Serag, 2020). Modern treatments for GC have been rapidly developed, mainly involving surgery combined with chemotherapy, radiotherapy, and targeted therapy (Johnston and Beckman, 2019; Joshi and Badgwell, 2021). However, challenges such as incomplete treatment, serious side effects, recurrence, and early metastasis still exist (Sexton et al., 2020), and these factors seriously affect the overall survival rate of patients with GC. Therefore, the discovery of new therapeutic treatments for GC is of great significance.

Increasing evidence suggests that many cancer chemotherapeutic drugs originate from natural product analogs (Yuan et al., 2017; Pham et al., 2019; Atanasov et al., 2021). However, a historical analysis of natural product drug discovery indicates that it is difficult to directly develop unmodified natural products. This is mainly due to the insufficient activity, complex structure, and high toxicity of natural products (Thomford et al., 2018; Sharifi-Rad et al., 2019). Therefore, it is necessary to optimize the structure of natural products by developing analogs to improve their pharmacological activity and druggability. The natural product evodiamine (EVO) is a quinolizidine alkaloid extracted from the traditional Chinese medicine Wu-Chu-Yu, which has been used to treat spleen and stomach diseases for thousands of years (Wang et al., 2020). Pharmacological activity studies have shown that EVO can inhibit the proliferation and apoptosis of various tumor cells, including GC cells (Huang et al., 2011; Rasul et al., 2012; Yang et al., 2014; Shen et al., 2015).

Recently, derivatives of EVO have been reported to exhibit anti-GC activity against tumor cells *in vitro* and *in vivo* (Hu et al., 2018; Liang et al., 2022a; Liang et al., 2022b; Hao et al., 2022). Hao et al. (2022) synthesized several N14 phenyl-substituted EVO derivatives and investigated their structure–activity relationships. They found that an N14 3-fluorinated phenyl-substituted EVO derivative exhibited good inhibitory activity against a gastric carcinoma cell line. Subsequently, introducing substituents into the A and E rings could improve the anti-GC activity of the N14 3-fluorinated phenyl-substituted EVO derivatives (Liang et al., 2022a; 2022b). Through systematic structural optimization and structure–activity relationship analysis, two EVO derivatives were found which were highly active against GC. In previous reports, EVO derivatives have been shown to possess topoisomerase 1 (TOP1) inhibitory activity (Hu et al., 2017; Chen et al., 2019; Chen et al., 2021). Therefore, the N14 phenyl-substituted EVO derivatives that showed good anti-GC activity were also confirmed to have inhibitory effects on TOP1. However, to the best of our knowledge, the inhibitory mechanisms of EVO derivatives against TOP1 have not yet been studied at the molecular level. Detailed investigation of the interactions between EVO derivatives and TOP1 can elucidate these inhibition mechanisms and provide a structural basis for the discovery and development of more effective TOP1 inhibitors.

Topoisomerases are a class of important enzymes that are widely present in organisms (Capranico et al., 2017). They are essential for cell DNA replication and transcription. During DNA replication, reverse rotation generates entanglements, as well as positive and negative supercoiling. The function of topoisomerases is to relax the DNA by releasing the torsional strain caused by supercoiling. As a result, topoisomerases can alter DNA topology by breaking and reconnecting the DNA strands (Champoux, 2001; Robert et al., 2016). The participation of topoisomerases in DNA cleavage and reconnection is essential to ensure normal replication, as they facilitate the unwinding, replication, and transcription of DNA. Currently, human topoisomerases are composed of six subcategories: TOP1, TOP1MT, TOP2A, TOP2B, TOP3A, and TOP3B (Pommier et al., 2022). Among these, TOP1 is a widely studied target of anticancer drugs.

TOP1 is overexpressed in several types of cancer cells, including GC (Selas et al., 2021). The content of TOP1 is much higher in cancer cells than in normal tissue, especially at the G2/M stage (Pommier et al., 2016). Consequently, cancer cells are highly sensitive to TOP1 inhibitors. Currently, nearly 50% of clinical cancer treatments rely on the use of one or more TOP1 inhibitors (Talukdar, 2022). The inhibitors of TOP1 can be classified into two categories: suppressors and poisons. Suppressors kill cells by hindering the catalytic function of TOP1. Poisons kill cells by capturing the cleavable DNA–TOP1 complex, forming a “road blocker” that prevents replication (Martín-Encinas et al., 2022). Camptothecin and its analogs, such as irinotecan (camptothecin-11) and topotecan, are currently widely used as TOP1 poisons in clinical practice, and they are also the most classic TOP1-specific inhibitors. Although camptothecin and its derivatives have achieved very good effects for cancer treatment, they have some serious shortcomings such as poor stability, short efficacy, susceptibility to drug resistance, and significant toxic side effects (Wang et al., 2023). These have prompted researchers to strive for more effective “non-camptothecin” TOP1 inhibitors as anticancer agents. EVO is a non-camptothecin TOP1 inhibitor that was identified through structure-based virtual screening (Dong et al., 2010) and biological assessment (Chan et al., 2009).

The crystal structures of human TOP1 in covalent and noncovalent complexes with 22-base pair duplex DNA have been reported (Redinbo et al., 1998). The enzyme contains 765 amino acid residues and comprises four domains: the N-terminal, core, linker, and C-terminal domains. The N-terminal domain consists of residues 1–124. The core domain is composed of residues 215–635, which can be further divided into three subdomains. Subdomain 3 contains the catalytic residues Arg488, Lys532, Arg590, and His632. The linker region contains residues 636–712. Amino acids 713–765 constitute the C-terminal domain and include the catalytic residue Tyr723. The core and C-terminal domains are closely connected through the linker region. These three regions form the main structure of the enzyme (Stewart et al., 1998). The enzyme structure provides a good structural model for a deep study on the molecular mechanism of TOP1 inhibition.

Computational approaches have significant advantages over traditional drug discovery in the design and discovery of novel anticancer drugs. They can improve the efficiency and accuracy of drug discovery and then quickly find the most effective drug candidate molecules. The process is short and simple, with a high success rate for new drugs. Computational methods have been successfully applied to the discovery and development of some new anticancer drugs such as sorafenib, lapatinib, and crizotinib (Cui et al., 2020). Molecular docking is a powerful method used to predict the interactions between the ligand and its receptor. Molecular docking enables the screening of compounds with potential anticancer effects based on the binding affinity between a ligand and a cancer-related target. This is helpful to reduce the number of compounds that need to be measured or synthesized *in vitro* and *in vivo* (Iwaloye et al., 2023). Molecular dynamics (MD) simulations can provide detailed structural insights into drug-target molecular interactions and can simulate the dynamic behavior of the drug-target complex. This helps explain the flexibility of receptors in the drug recognition process (Razia et al., 2023). The binding free energy can quantitatively characterize the binding strengths and analyze critical interactions of the drug-target complex (Wang et al., 2019).

**FIGURE 1:**
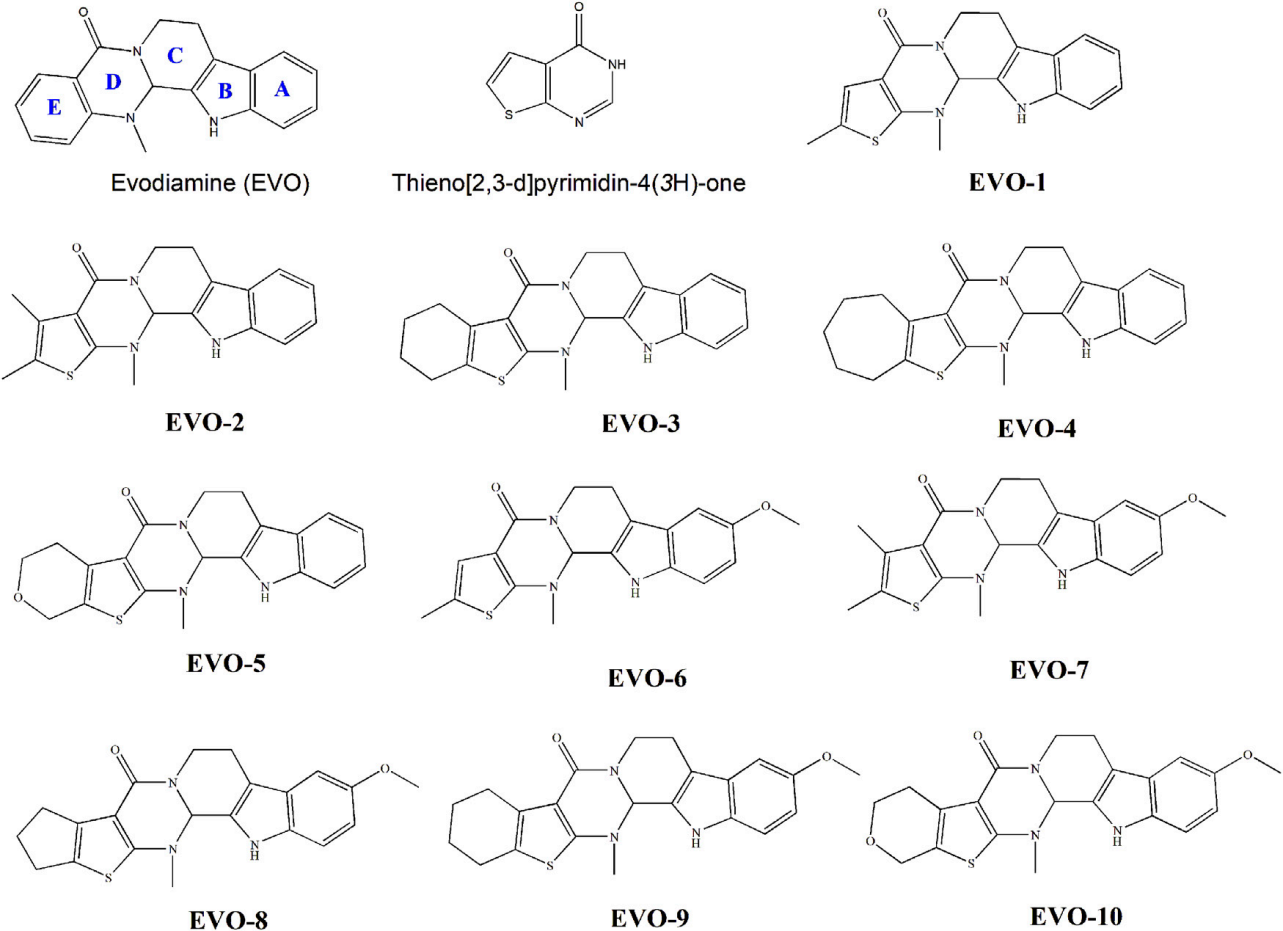
Molecular structures of evodiamine, thieno [2,3-d] pyrimidin-4(*3H*)-one, and evodiamine derivatives.

Recently, we synthesized several hybrids of EVO and thieno [2,3-d] pyrimidinones (Nie et al., 2020). The anti-proliferative activities of these compounds on tumor cells were evaluated. The result showed that most of these compounds exhibited moderate anticancer activity. Their structures are shown in Figure 1. These compounds have the potential to be developed as novel antitumor lead compounds. However, we have not yet studied the inhibitory effects of these compounds on GC cells. The inhibition mechanism of these compounds on TOP1 remains unclear, which hinders their use as lead compounds to design TOP1 inhibitors. As part of our ongoing research, we investigated the anti-GC activity of these EVO derivatives, based on the clinical efficacy of Wu-Chu-Yu for the treatment of gastrointestinal disorders. The structure–activity relationships and TOP1 inhibition mechanisms of these EVO derivatives were elucidated from a computational perspective.

## 2 Materials and methods

### 2.1 Cell culture and MTT assay

GC SGC7901 cells in the logarithmic phase were harvested and counted using a hemocytometer to maintain a cell density of 5 × 10^3^ per well. The 96-well plates were incubated at 37°C with 5% CO_2_ for 24 h before further use. For the methyl thiazolyl tetrazolium (MTT) assay, different drug concentrations were added to SGC7901 cells in the logarithmic growth phase. Two hundred microliters of drug-containing medium or blank medium was added to each well. For each drug group, six duplicate wells were used. The absorbance (optical density, OD) of each well was measured at 450 nm with a microplate reader. The OD value was used to calculate the survival rate of SGC7901 cells with each drug, and the IC_50_ value was calculated using GraphPad Prism 9.0.

### 2.2 Density functional theory calculations

To reveal the relationship between electronic structure and anti-GC activity, density functional theory (DFT) studies of quantum chemistry were carried out on the ten EVO derivatives. First, the molecular structures of the EVO derivatives were fully optimized to obtain equilibrium geometries at the B3LYP/6-311++G^**^ level of theory. The frequency of the optimized geometry was then calculated to identify the nature of the stationary points. The results showed that there were no imaginary frequencies in the optimized structures of the EVO derivatives. This indicated that the optimized geometries corresponded to stationary points and minima. Finally, natural bond orbital charges and frontier molecular orbitals (FMOs) were calculated at the same level. All DFT calculations were performed using Gaussian 09 software (Frisch et al., 2009).

### 2.3 Molecular docking

The initial structure of the receptor–ligand complex was obtained from molecular docking using the AUTODOCK 4.2 program (Morris et al., 2009). Ligand models were obtained from the optimized geometries of the DFT calculations. The initial receptor model was extracted from the X-ray crystal structure of TOP1 in complex with the inhibitor, topotecan, obtained from the Protein Data Bank (PDB:1K4T) (Staker et al., 2002). The crystal structure had 765 amino acid residues and a resolution of 2.10 Å. The ligand and receptor files were examined and prepared using AutoDock Tools 1.5.6.

The original binding inhibitor (topotecan) and crystal water molecules were deleted and polar hydrogen atoms and Gasteiger charges were added to the initial receptor structure. Nonpolar hydrogens were merged. For each docking ligand, rotatable bonds were defined. The receptor is considered a rigid molecule, whereas the ligand is flexible. A grid of 44 × 44 × 44 with 0.375 Å grid spacing was defined as the docking box for each complex. Docking studies were carried out using the Lamarckian genetic algorithm (Fuhrmann et al., 2010) in AutoDock 4.2.

For each system, one hundred conformations were generated. Finally, the optimal conformation with the best rational orientation in the DNA–TOP1 pocket was selected from the docking experiment.

### 2.4 MD simulations

The optimal docking conformation for the complex was used as the initial structure for the MD simulations. General AMBER force field (GAFF, version 2) (Wang et al., 2004) was employed to describe the force field for the ligand molecules. The parameters for the ligand were generated by the antechamber module. The topology parameters for TOP1 and the DNA were created using the Amber ff19SB force field (Tian et al., 2020).

For each system, a total of 100 ns MD simulations were performed to collect data. The system was solvated in a water box using an explicit TIP3P water model. The marginal distance of the water box was set at 15 Å to guarantee that the system was completely immersed in the solution. The total charges of the system were calculated and Na^+^ was added to neutralize them. First, to avoid unfavorable interactions caused by solvents and ions, the system was subjected to minimization using steepest descent and conjugate gradient methods. Subsequently, the complex system was heated from 0 to 300 K in an NVT ensemble using Langevin dynamics (Feller et al., 1995). The NPT ensemble was subjected to equilibrium for 50 ps at 300 K at a constant pressure of 1 atm. Finally, 10,000 frames were extracted from a total of 100 ns of simulations as the average conformation of the MD equilibrium for statistical analysis. All dynamic simulations were performed using the PMEMD CUDA version of AMBER 20 (Case et al., 2020). The MD trajectory data were analyzed by the CPPTRAJ module (Roe and Cheatham, 2018; Cheatham et al., 2019).

### 2.5 Binding free energy calculation

The molecular mechanics generalized Born surface area (MM/GBSA) method (Srinivasan et al., 1998; Lee et al., 2004) was used to calculate the binding free energy between the two most active EVO derivatives and TOP1. This is an effective method to quantitively evaluate the binding affinity between a ligand and receptor. The energy term for each complex was calculated by averaging over 2,000 frames of the last 20 ns of an MD trajectory. To evaluate the contribution of each residue, the total binding free energy between DNA–TOP1 and the ligand was decomposed using MM/GBSA binding free energy decomposition (Gaillard and Simonson, 2014). The contribution of entropy was not included in this approach. The MMPBA.py (Case et al., 2020) program was used to calculate free energy.

## 3 Results and discussion

### 3.1 Anti-GC activity of EVO derivatives

An MTT assay was used to investigate the proliferative inhibitory effects of different concentrations of the target compounds on the GC SGC7901 tumor cell line, with EVO as the parent compound. The growth inhibition rate of the compound on tumor cells was determined by calculating the percentage of dead cells (Supplementary Figure S1). As shown in Supplementary Figure S1, the inhibition rate of these 10 derivatives gradually increased with increasing drug concentration. They displayed a concentration-dependent relationship. At 200 μmol·L^−1^, the inhibition rate of each compound reached its maximum. The inhibition rates of the EVO derivatives in GC SGC7901 cells were higher than that of EVO, except EVO-5 and EVO-8. The order of the anti-GC activity of the ten tested derivatives was as follows: EVO-8 < EVO-5 < EVO-10 < EVO-3 < EVO-7 < EVO-2 < EVO-9 < EVO-4 < EVO-6 < EVO-1. The inhibition rates of EVO-1 and EVO-6 were 81.17% ± 5.08% and 80.92% ± 2.75%, respectively, indicating that among these 10 derivatives, EVO-1 and EVO-6 had the strongest anti-GC activity. Therefore, EVO-1 and EVO-6 were selected for subsequent analysis of the TOP1 inhibition mechanisms.

### 3.2 DFT calculation studies

The chemical structure of a drug determines its physicochemical properties and directly affects the absorption, distribution, metabolism, and excretion of drug molecules in the body (Nehra et al., 2022). The relationship between their structure and activity is of great significance for the design and development of drugs. To investigate the relationship between structure and activity, we performed DFT calculations on the 10 EVO derivatives. The molecular structures of these 10 EVO derivatives were fully optimized using the DFT method at the level of B3LYP/6-311++G^**^. The equilibrium geometries and atom numbers of these optimized derivatives are shown in Supplementary Figure S2. For compound EVO-4, the optimized structure was consistent with the X-ray structure (Nie et al., 2020). The optimized geometries of other derivatives were almost nonplanar, similar to the optimized geometry of EVO (Liu et al., 2022).

Compounds EVO-1 to EVO-5 underwent alkyl substitution on the thiophene ring. The anti-GC activity of these compounds decreased with the increasing steric hindrance of the alkyl substituent on the thiophene ring. Based on the structures of compounds EVO-1 to EVO-5, compounds EVO-6 to EVO-10 introduced a methoxy group at position 10 of the indole ring. When methoxy substituents were introduced at position 10 of the A ring, anti-GC activity decreased to a certain extent. For example, the activity of EVO-6 was lower than that of EVO-1. This indicates that the methoxy substituent at position 10 of the A ring decreased the anti-GC activity to some extent. One previous study also showed that 10-methoxy substitution reduces the antitumor activity of EVO derivatives (Fan et al., 2021). However, a more recent study (Liang et al., 2022a) showed that antitumor activity was improved to some extent after the introduction of methoxy, methyl, and chlorine substituents at position 10 of the A ring. In particular, 10-chlorine-substituted derivatives showed significant antitumor activity against different GC cell lines. However, by comprehensively considering both antitumor activity and toxicity, 10-methyl was found to be more suitable. Among these 10 derivatives, EVO-1 and EVO-6 showed the greatest inhibitory activity on the proliferation of SGC7901 cells. This may indicate that a less bulky substitute group results in better anti-GC activity. In addition, di-substitution between the A ring and thiophene ring may have a synergistic effect. EVO-1 and EVO-6 may serve as promising compounds for the development of potential anti-GC agents.

#### 3.2.1 Natural population charge

The charge of drug molecules directly affects their active sites and interactions with other small molecules or biomacromolecules, especially their interactions with their targets *in vivo* (Mohamed et al., 2020). Therefore, charge analysis can provide theoretical guidance for the further structural modification of drug molecules. The charges of the main atoms of EVO-1 to EVO-10 are listed in Table 1. The lower the atomic charge, the stronger the electrophilic ability; in contrast, the higher the charge, the stronger the nucleophilic ability.

**TABLE 1:** Natural population charge of the atoms of compounds 1–10.

Atom	EVO-1	EVO-2	EVO-3	EVO-4	EVO-5	EVO-6	EVO-7	EVO-8	EVO-9	EVO-10
1N	−0.547	−0.547	−0.547	−0.547	−0.547	−0.549	−0.549	−0.549	−0.549	−0.549
2C	0.132	0.134	0.134	0.134	0.132	0.137	0.138	0.138	0.139	0.137
3C	0.228	0.227	0.227	0.226	0.227	0.229	0.228	0.228	0.228	0.227
4N	**−0.552**	**−0.552**	**−0.552**	**−0.551**	**−0.550**	**−0.548**	**−0.548**	**−0.548**	**−0.548**	**−0.547**
5C	−0.178	−0.178	−0.178	−0.178	−0.178	−0.167	−0.167	−0.167	−0.167	−0.168
6C	−0.357	−0.357	−0.357	−0.358	−0.358	−0.394	−0.394	−0.394	−0.394	−0.394
7C	−0.096	−0.096	−0.097	−0.097	−0.096	−0.087	−0.087	−0.087	−0.088	−0.087
8C	−0.109	−0.109	−0.109	−0.109	−0.109	−0.064	−0.063	−0.064	−0.063	−0.064
9C	**−0.105**	−0.105	−0.105	−0.105	−0.105	−0.219	**−0.219**	−0.219	−0.219	−0.218
10C	**−0.258**	**−0.258**	**−0.259**	**−0.259**	**−0.258**	**0.303**	**0.302**	**0.303**	**0.302**	**0.302**
11C	−0.203	−0.204	−0.204	−0.204	−0.203	−0.282	−0.282	−0.282	−0.282	−0.282
12C	−0.234	−0.234	−0.234	−0.234	−0.234	−0.214	−0.214	−0.214	−0.214	−0.215
13C	0.160	0.160	0.160	0.160	0.160	0.141	0.141	0.141	0.141	0.142
14N	**−0.559**	**−0.559**	**−0.560**	**−0.560**	**−0.558**	**−0.558**	**−0.559**	**−0.560**	**−0.559**	**−0.559**
15C	**0.686**	**0.687**	**0.690**	**0.691**	**0.690**	**0.686**	**0.687**	**0.690**	**0.690**	**0.691**
16O	**−0.623**	**−0.629**	**−0.631**	**−0.635**	**−0.630**	**−0.623**	**−0.630**	**−0.625**	**−0.631**	**−0.631**
17C	−0.361	−0.360	−0.360	−0.361	−0.360	−0.361	−0.360	−0.360	−0.360	−0.362
31C	−0.179	−0.185	−0.179	−0.172	−0.225	−0.179	−0.185	−0.211	−0.179	−0.225
32C	−0.220	−0.049	−0.044	−0.043	−0.039	−0.220	−0.049	−0.037	−0.044	−0.039
33C	−0.217	−0.213	−0.216	−0.212	−0.216	−0.217	−0.213	−0.220	−0.216	−0.216
34C	0.056	0.060	0.060	0.052	0.065	0.057	0.060	0.066	0.060	0.067
35S	**0.403**	**0.407**	**0.416**	**0.420**	**0.422**	**0.403**	**0.406**	**0.426**	**0.415**	**0.421**
36C	**−0.600**	**−0.600**	**−0.410**	**−0.417**	**−0.034**	**−0.600**	**−0.600**	**−0.386**	**−0.410**	**−0.034**
37C	—	−0.575	−0.385	−0.403	−0.409	—	−0.575	−0.371	−0.385	−0.409
26O	—	—	—	—	—	**−0.551**	−0.551	−0.551	−0.551	−0.549

The bold values describe the important results.

As shown in Table 1, the charge distributions of EVO-1 to EVO-10 were generally very similar. The 1N, 4N, 14N, and 16O atoms have more negative charges, indicating that these are electrophilic active sites and that electrophilic groups can be introduced into them during structural modification. The 15C and 35S atoms have the most positive charges, indicating that these two positions are nucleophilic active sites prone to nucleophilic substitution. Nucleophilic reagents can be introduced at these two positions during structural modification. In comparison, owing to the introduction of the methoxy group, the charge on the 10C atom changed from a negative value for EVO-1 to EVO-5 to a positive value for EVO-6 to EVO-10. This suggests that EVO-1 to EVO-5 may interact with the active site residues of the receptor as an electron donor, while EVO-6 to EVO-10 may interact with the active site residues of the receptor as an electron acceptor. It is inferred that the interaction mode between EVO-1 and TOP1 may be different from that between EVO-6 and TOP1.

#### 3.2.2 Molecular electrostatic potential

**FIGURE 2:**
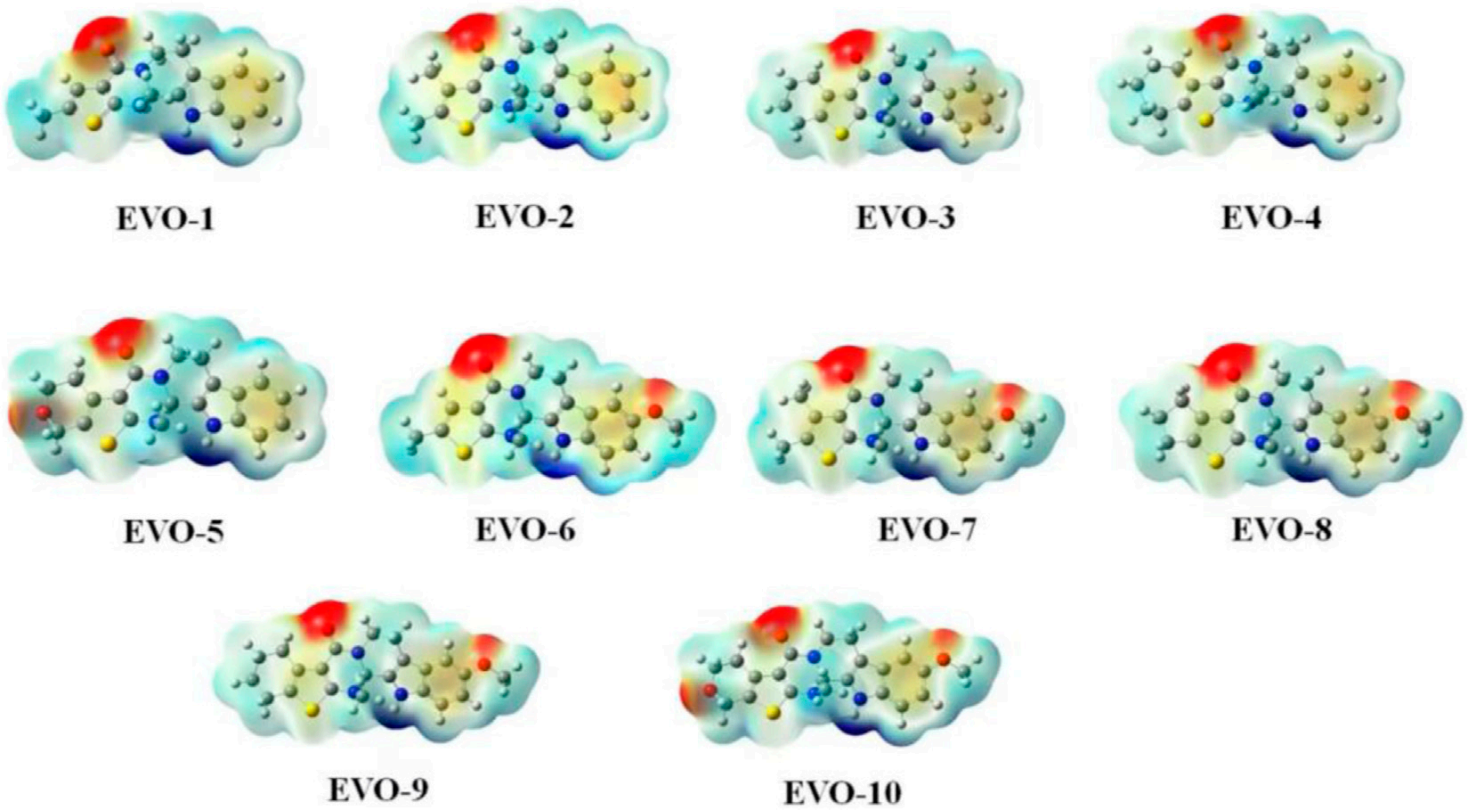
Molecular electrostatic potential maps for compounds EVO-1 to EVO-10. Mapping of molecular electrostatic potential on the iso-surface of electron density with an iso-value of 0.001. The molecular electrostatic potential ranges from −0.04 (red) to 0.04 (blue). Values are in atomic units.

Molecular electrostatic potential (MEP) is of great significance for investigating electrostatic interactions between small molecules and protein molecules. The MEP can also be used to predict the active sites and positions for molecular recognition (Shafieyoon et al., 2019). The calculated MEPs for these 10 compounds are plotted in Figure 2. Positive MEPs were concentrated on the hydrogen atom of the indole ring, illustrating that this is the preferred hydrogen bond donor. Negative MEPs were distributed around the carbonyl oxygen atoms. In addition, for EVO-6 to EVO-10, a negative MEP was observed for the oxygen atom of the methoxy group. This may demonstrate that the oxygen atoms of the carbonyl and methoxy groups are the preferred hydrogen bond acceptors.

#### 3.2.3 Frontier molecular orbitals

FMOs are molecular orbitals that are located in the outermost layer of electrons in molecules. These orbitals are closely related to the chemical properties of molecules and are typically used to depict the properties of inhibitors and their possible interactions with receptors in computer aided drug design. They are composed of the highest occupied molecular orbital (HOMO) and lowest unoccupied molecular orbital (LUMO) of the electrons.

The higher the HOMO energy, the easier it is for a molecule to lose its outermost electrons. In contrast, the lower the LUMO energy, the easier it is for a molecule to obtain electrons. Therefore, the activity and stability of the molecule are affected by the energy gap between the HOMO and LUMO. The calculated HOMO and LUMO energies, as well as the energy gaps, of these 10 compounds in the gas phase are listed in Table 2. From the perspective of the energy gaps, the activity order of the 10 derivatives was as follows: EVO-2 < EVO-4 < EVO-3 < EVO-1 < EVO-5 < EVO-9 < EVO-7 < EVO-8 < EVO-6 < EVO-10. These calculated results are slightly different from those of the MTT experiment. This may be because the DFT calculations for these derivatives were performed in the ideal gas phase. To simulate the real physiological environment, it will be necessary to consider the protein environment in future studies.

**TABLE 2:** HOMO and LUMO energies and the energy gap between HOMO and LUMO for the derivatives of EVO (units: eV).

Compound	EVO-1	EVO-2	EVO-3	EVO-4	EVO-5	EVO-6	EVO-7	EVO-8	EVO-9	EVO-10
*E*_(HOMO)_	−5.95	−5.93	−5.90	−5.89	−6.00	−5.67	−5.66	−5.66	−5.65	−5.71
*E*_(LUMO)_	−1.22	−1.17	−1.16	−1.15	−1.30	−1.19	−1.15	−1.17	−1.14	−1.31
Δ*E*_(LUMO−HOMO)_	4.73	4.75	4.74	4.74	4.70	4.48	4.51	4.49	4.51	4.41

HOMO, highest occupied molecular orbital; LUMO, lowest unoccupied molecular orbital.

Based on FMO theory, FMOs play a crucial role in predicting the interactions between the inhibitor and receptor. The LUMO of the inhibitor can interact with the HOMO of the receptor. Similarly, the HOMO of the inhibitor can interact with the LUMO of the receptor. The HOMOs and LUMOs of the 10 EVO derivatives are shown in Supplementary Figure S3. The red and green colors represent the positive and negative orbitals, respectively. The iso-density surface plots of the 10 derivatives were similar. The HOMOs were mainly localized on rings A and B, except in EVO-2, EVO-3, and EVO-4. However, the LUMOs were delocalized over the entire system.

### 3.3 Molecular docking

**FIGURE 3:**
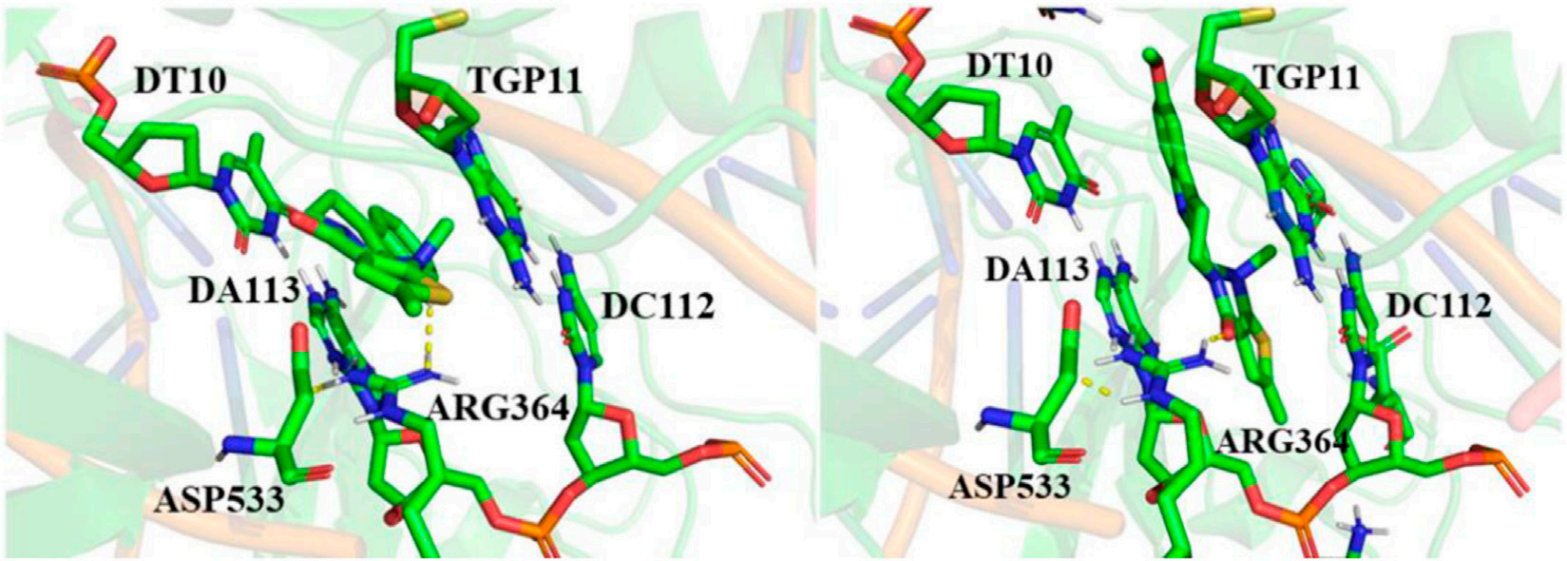
Binding conformations of EVO-1 (left) and EVO-6 (right) with topoisomerase 1 (TOP1). Hydrogen bond interactions between the small molecules and receptors are indicated by yellow dotted lines.

Molecular docking is an important method for structure-based drug design. It can predict the binding mode and affinity between small ligand molecules and receptor biomolecules (Meng et al., 2011; Luca and Giulio, 2019). To gain a deep understanding of the anti-GC activity of compounds EVO-1 and EVO-6, we utilized molecular docking techniques to investigate the ligand–receptor interactions of EVO-1 and EVO-6 with the known anticancer target TOP1. As shown in Figure 3, we plotted the binding models of EVO-1 and EVO-6 with TOP1. The binding conformations of EVO-1 and EVO-6 with TOP1 were very similar, which is consistent with the previous predicted binding mode of EVO with TOP1 (Liu et al., 2022). Despite the nonplanar structure of EVO and its derivatives, they can still bind to the active site of TOP1. Their orientation in the active site cavity is almost perpendicular to the main axis of the DNA and parallel to the base pairs. In contrast with the binding pattern of topotecan in the crystal structure (Staker et al., 2002), the EVO derivatives bind at the entrance of the active cavity. This suggests that pharmaceutical chemists can introduce other functional groups to occupy the active cavity.

The ligand intercalated at the site of DNA cleavage, with the A ring directed toward the major groove and the E ring pointing toward the minor groove. Base-stacking interactions occurred between the ligand and DNA base pairs. The long axis of the ligand molecule was parallel to the axis of base pairing. This intercalation-binding mode may explain how EVO-1 and EVO-6 specifically block DNA relaxation. Therefore, they could have inhibitory effects on TOP1, exerting anti-GC activity. This implies that EVO-1 and EVO-6 have the potential to be developed as lead compounds against GC.

The docking model also predicted that only one direct hydrogen bond was formed between the enzyme and EVO-1. The distance between the carboxyl oxygen of Asp533 and the carbonyl oxygen of EVO-1 is 3.2 Å, and they can therefore form a hydrogen bond. In contrast, this hydrogen bond is replaced by the NH of Arg364 and the carbonyl of EVO-6. This hydrogen-bond interaction can be interpreted by MEPs. The MEP around the carbonyl oxygen atom is negative, which facilitates formation of the hydrogen bond. The importance of the carbonyl group is also supported by structure–activity studies of EVO (Dong et al., 2012). For example, when the carbonyl O is replaced by S, the antitumor activity of the molecule is weakened. This indicates that hydrogen bonding between the ligand and Asp533 is important for antitumor activity. This hydrogen-bonding interaction needs to be maintained or replaced by new hydrogen bonding during the modification of the EVO structure. Asp533 was also coordinated to the e-nitrogen of Arg364. The distance between the hydrogen atom of the e-nitrogen of Arg364 and the carbonyl group of Asp533 is 2.2 Å for EVO-1 and 1.9 Å for EVO-6. This demonstrates that Arg364 and Asp533 are critical for ligand binding.

Molecular docking revealed that TOP1, DNA, and EVOs formed ternary complexes. The ternary TOP1–DNA–ligand complex is stabilized by base-stacking interactions and several protein–DNA interactions. The binding energy of EVO-1 with TOP1 was −7.28 kcal·mol^−1^, which was lower than that for EVO-6 (−7.16 kcal·mol^−1^). This indicates that EVO-1 has a better anti-GC effect than EVO-6. Thus, it was further demonstrated that EVO-1 can be used as a potential anti-GC candidate.

### 3.4 MD simulations

**FIGURE 4:**
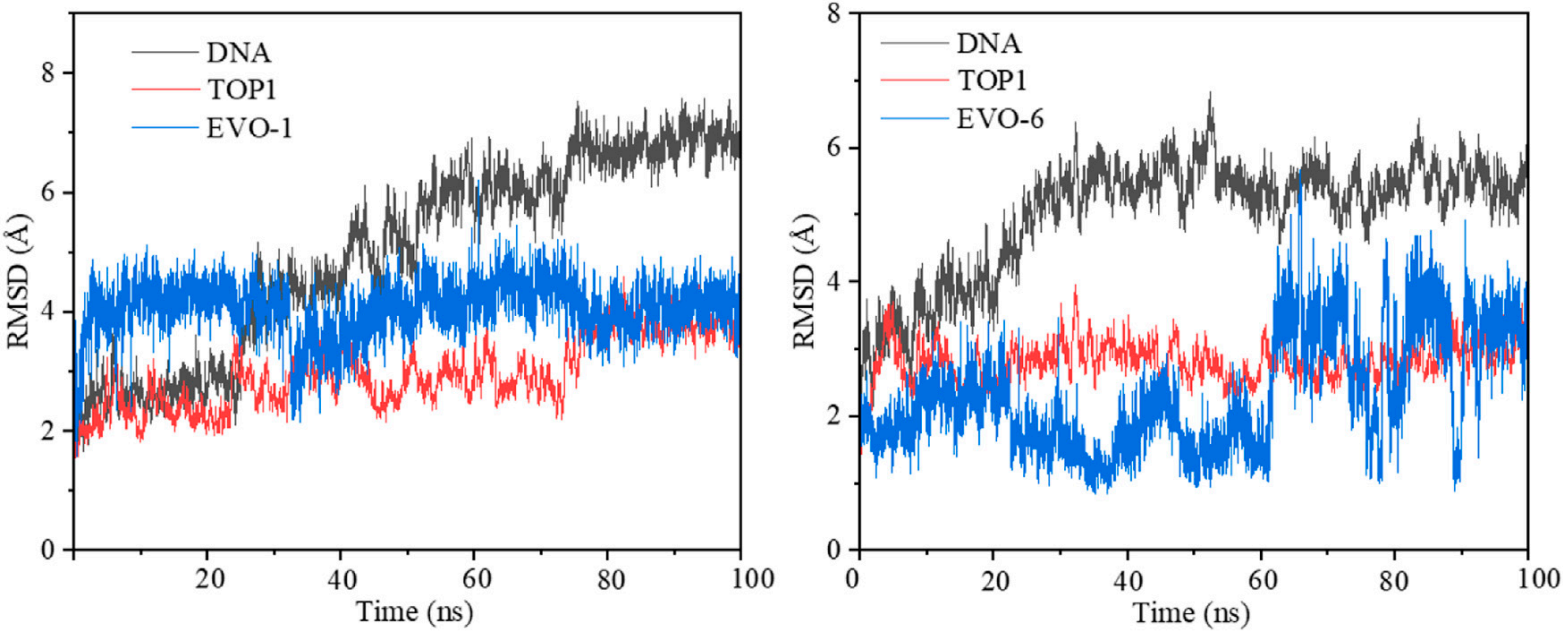
Root-mean-square deviation (RMSD) value of the DNA and the heavy atoms of proteins and inhibitors along 100 ns molecular dynamics simulations for the TOP1/EVO-1 (left) and TOP1/EVO-6 systems (right). TOP1, topoisomerase 1.

To simulate the dynamic characteristics of the ternary TOP1–DNA–ligand complex, MD simulation studies are required. A total of 100 ns MD simulations were performed to estimate the stability of the TOP1/EVO-1 and TOP1/EVO-6 systems. The root-mean-square deviation values of the DNA and the heavy atoms of the proteins and inhibitors for the TOP1/EVO-1 and TOP1/EVO-6 systems are shown in Figure 4. The results indicate that fluctuations in the protein, DNA, and inhibitor structures were relatively small during the MD simulation process. This illustrates that a stable TOP1–DNA–ligand ternary complex was formed. In other words, the ligand can stably bind at the cleaved DNA-binding site. This stable ternary complex may lead to DNA damage, which can, in turn, induce cancer cell apoptosis. Thus, EVO-1 and EVO-6 can exert anti-GC effects by stabilizing the covalent TOP1–DNA complex. The two TOP1–DNA–ligand ternary complex structures were used for subsequent analyses.

The fluctuations of each atom relative to its average position were calculated by root-mean-square fluctuation (RMSF), which can characterize the average effect of structural changes over time and the flexibility of each region of the protein. During MD simulations the stability of specific residues can be quantified by RMSF. RMSF was used to examine whether the simulation results were consistent with the crystal structure. The RMSF values for the TOP1/EVO-1 and TOP1/EVO-6 systems are illustrated in Supplementary Figure S4. The residues at positions 235–248, 287–299, 283–338, 388–400, 510–524, 635–646, and 656–697 of TOP1 exhibited a certain degree of fluctuation (RMSFs >1.50 Å). This demonstrates that the core domain of the enzyme is flexible. The linker region containing amino acids 636–712 showed large fluctuations with RMSFs >2.50 Å. The calculated results support that the linker region has little influence on the affinity of ligand binding to TOP1 (Stewart et al., 1997).

During MD simulation, the flexibility and extensibility of a ligand binding inside the pocket can be described by the radius of gyration. In Supplementary Figures S5, S6, we plotted the radius of gyration of the protein, DNA, and ligand for the TOP1/EVO-1 and TOP1/EVO-6 systems, respectively. It is evident that these two systems reached a stable state in the 100 ns MD simulations. The surfaces of the protein, DNA, and ligands of the TOP1/EVO-1 and TOP1/EVO-6 systems are shown in Supplementary Figures S7, S8. The surface of the protein did not show any significant fluctuations in the binding pocket throughout MD simulation. The small ligand surface area indicated that it was completely enclosed in the cavity of the active site. A clamp around the DNA was formed between the C-terminal domain and the core domain (Redinbo et al., 1998; Chillemi et al., 2001). Thus, most of the solvent-accessible surfaces of the ligands were covered by TOP1.

For each TOP1–DNA–ligand system, snapshots were taken from MD trajectories at intervals of 10 ns. They were aligned as shown in Supplementary Figures S9, S10. The conformation of the ligand underwent minor changes, while the conformation of the protein showed little change except in the linker region. This phenomenon can be explained by the significant RMSF fluctuations of the linker region. A stable ternary complex was formed between the ligand and the covalent complex of TOP1–DNA. A close examination of the ternary complex revealed that there was sufficient space within the binding pocket to allow the introduction of other groups.

### 3.5 Binding free energies

The binding free energy can be used to quantitatively predict the binding affinity between the ligand and receptor. The binding free energies of the two complex systems were calculated by MM/GBSA and are summarized in Supplementary Tables S1, S2. The calculated binding free energy was −38.33 kcal·mol^−1^ for the TOP1/EVO-1 system and −33.25 kcal·mol^−1^ for the TOP1/EVO-6 system. This can explain the activity results of the MTT assay at the molecular level, which showed that EVO-1 had a stronger anti-GC effect than EVO-6. The results of free energy calculations agree with those of molecular docking. The binding free energy results also indicated that EVO-1 is a promising candidate as a potential chemotherapeutic agent against GC. This conclusion is also consistent with the molecular docking results.

Meanwhile, the van der Waals (vdW) interactions for the TOP1/EVO-1 and TOP1/EVO-6 systems were −48.07 and −53.01 kcal·mol^−1^, respectively. This suggests that the vdW interactions are favorable for the formation of the ternary TOP1–DNA–ligand complex. The contributions of the electrostatic interactions to the binding free energies of EVO-1 and EVO-6 were −9.96 and 2.62 kcal·mol^−1^, respectively. The results indicate that, for the two binding models, the vdW interactions contributed more to the binding free energy than the electrostatic interactions. This suggests that vdW interactions play a crucial role in ligand binding with the covalent complex TOP1–DNA.

It is known that the polar (E_ele_ + E_GB_) and nonpolar (E_vdw_ + E_surf_) terms affect the binding of inhibitors to their targets. The polar contributions for TOP1/EVO-1 and TOP1/EVO-6 were 11.88 and 22.78 kcal·mol^−1^, respectively. The positive polar contribution values indicated that polar interactions were not favorable for binding between the ligand and receptor. Therefore, for the TOP1/EVO-1 and TOP1/EVO-6 systems, polar interactions were not favorable for ligand–receptor binding. This means that the lower the energy of the polar term, the more favorable the binding between the ligand and receptor. This can be used to explain why EVO-1 has stronger binding ability than EVO-6. Therefore, EVO-1 exhibited stronger anti-GC activity than EVO-6.

For TOP1/EVO-1 and TOP1/EVO-6, the total nonpolar values were −51.14 and −56.94 kcal·mol^−1^, respectively. This indicates that the nonpolar terms contribute significantly to the formation of ternary TOP1–DNA–ligand complexes. Thus, it can be inferred that the nonpolar term plays a dominant role in ligand binding. Hydrophobic interactions are the main determinant factors involved in the binding of EVO-1 and EVO-6 to TOP1–DNA.

### 3.6 Free energy decomposition

The main idea of free energy decomposition is to decompose the free energy of the receptor–ligand complex to each amino acid residue, thereby obtaining the energy contribution of each amino acid to the binding. The MM/GBSA decomposition protocol was employed to calculate the energy contribution of each amino acid in the binding complex (Supplementary Tables S3, S4). 5′-thio-2′-deoxyguanosine phosphonic acid (TGP) made a significant contribution to the complex, with energy values of −8.59 kcal·mol^−1^ for the EVO-1/TOP1 system and −4.35 kcal·mol^−1^ for the EVO-6/TOP1 system. This demonstrates that the Π–Π stacking interaction between TGP and the ligand is important for ligand binding.

For EVO-1/TOP1, residue Arg364 contributed −3.43 kcal·mol^−1^ because of the hydrogen bonding interaction; for EVO-6/TOP1, the hydrogen bonding interaction between Arg364 and EVO-6 contributed −0.84 kcal·mol^−1^. This indicates that the hydrogen bonding interaction between the ligand and Arg364 plays an important role in the binding models. Additionally, other hydrophobic residues such as DT10 and DA113 had substantial binding free energies. These residues are located at cleaved DNA-binding sites. Thus, DT10 and DA113 are key DNA residues for ligand binding with TOP1–DNA. These results explain the stronger anti-GC effect of EVO-1 than that of EVO-6.

## 4 Conclusion

The anti-GC activities and molecular mechanisms of EVO derivatives were studied using experimental and computational methods. The anti-proliferative activity of all the compounds against the GC cell line SGC-7901 was evaluated using MTT assays, with EVO as the parent compound. The results showed that EVO-1 and EVO-6 had better anti-GC activity than the parent compound.

The molecular structures and properties of EVO derivatives were optimized using DFT. The natural population charges, FMOs, and MEPs of these derivatives were used to analyze their structure–activity relationships. To understand the inhibitory mechanisms of EVO-1 and EVO-6 against the anticancer target TOP1, molecular docking, MD simulations, and free energy calculations were performed. The calculated results showed that EVO-1 and EVO-6 could bind to the cleaved DNA-binding sites, forming a stable TOP1–DNA–ligand ternary complex. Both EVO derivatives showed good inhibitory effects against TOP1, with binding free energies of −38.33 kcal·mol^−1^ and −33.25 kcal·mol^−1^, respectively. These results indicate that compounds EVO-1 and EVO-6 are potential lead compounds for anti-GC treatment and deserve further investigation. Meanwhile, novel TOP1 inhibitors may be developed for the treatment of GC in the future.

## Data Availability

The original contributions presented in the study are included in the article/Supplementary Material, further inquiries can be directed to the corresponding authors.
