# Supplementary material for: Experimental and computational study on anti-gastric cancer activity and mechanism of evodiamine derivatives
Source: Front Pharmacol. 2024 May 7;15:1380304. doi: 10.3389/fphar.2024.1380304 (PMC11113551; doi:10.3389/fphar.2024.1380304)
Supplement: Supplementary file 1 [file DataSheet1.docx]

Supplementary Material

Jingli Liu^1^, Yingying Xue^1^, Xinxin Yang^1^, Fei Yan^2^, Xu Long^1^, Hui Guo^1^, Hao Yan^1^, Guozheng Huang^3^, Jing Zhou^1*^, Yuping Tang^2*^

^1^ School of Pharmacy, Shaanxi University of Chinese Medicine, Xianyang, Shaanxi, People’s Republic of China

^2^ Key Laboratory of Shaanxi Administration of Traditional Chinese Medicine for TCM Compatibility, Shaanxi University of Chinese Medicine, Xianyang, Shaanxi, People’s Republic of China

^3^ College of Chemistry and Chemical Engineering, Anhui University of Technology, Ma'anshan, Anhui, People’s Republic of China

***** Jing Zhou and Yuping Tang
[2051124@sntcm.edu.cn](mailto:2051124@sntcm.edu.cn) (JZ); [yupingtang@sntcm.edu.cn](mailto:yupingtang@sntcm.edu.cn) (YT)


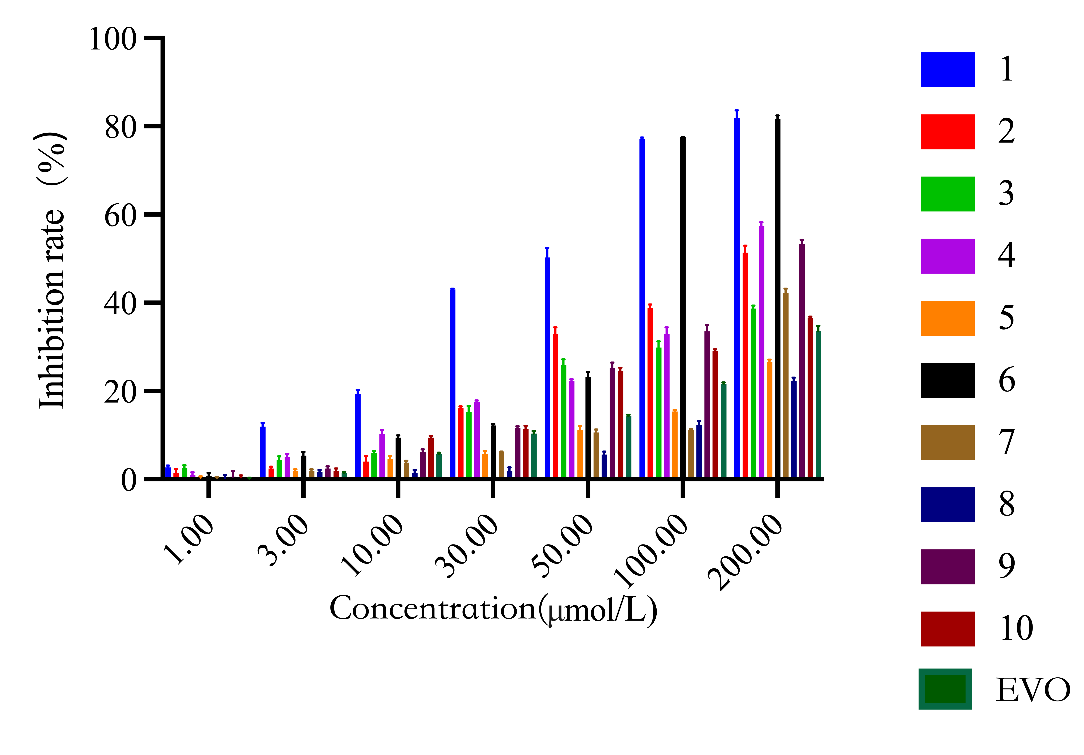


**Figure S1.** Antiproliferative effects of the derivatives of EVO on the gastric cancer SGC7901 tumor cells at different drug concentrations.


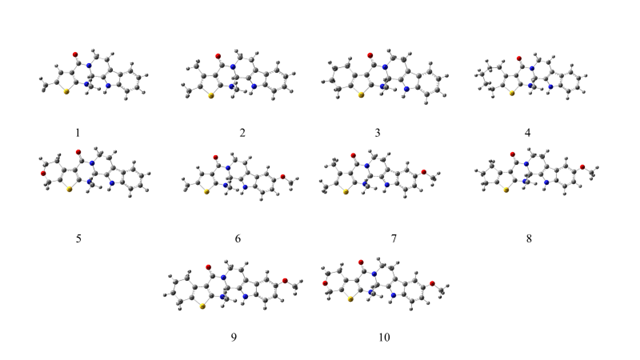


**Figure S2**. The optimized geometries and atom numbering of evodiamine derivatives (compounds labeled as 1-10)


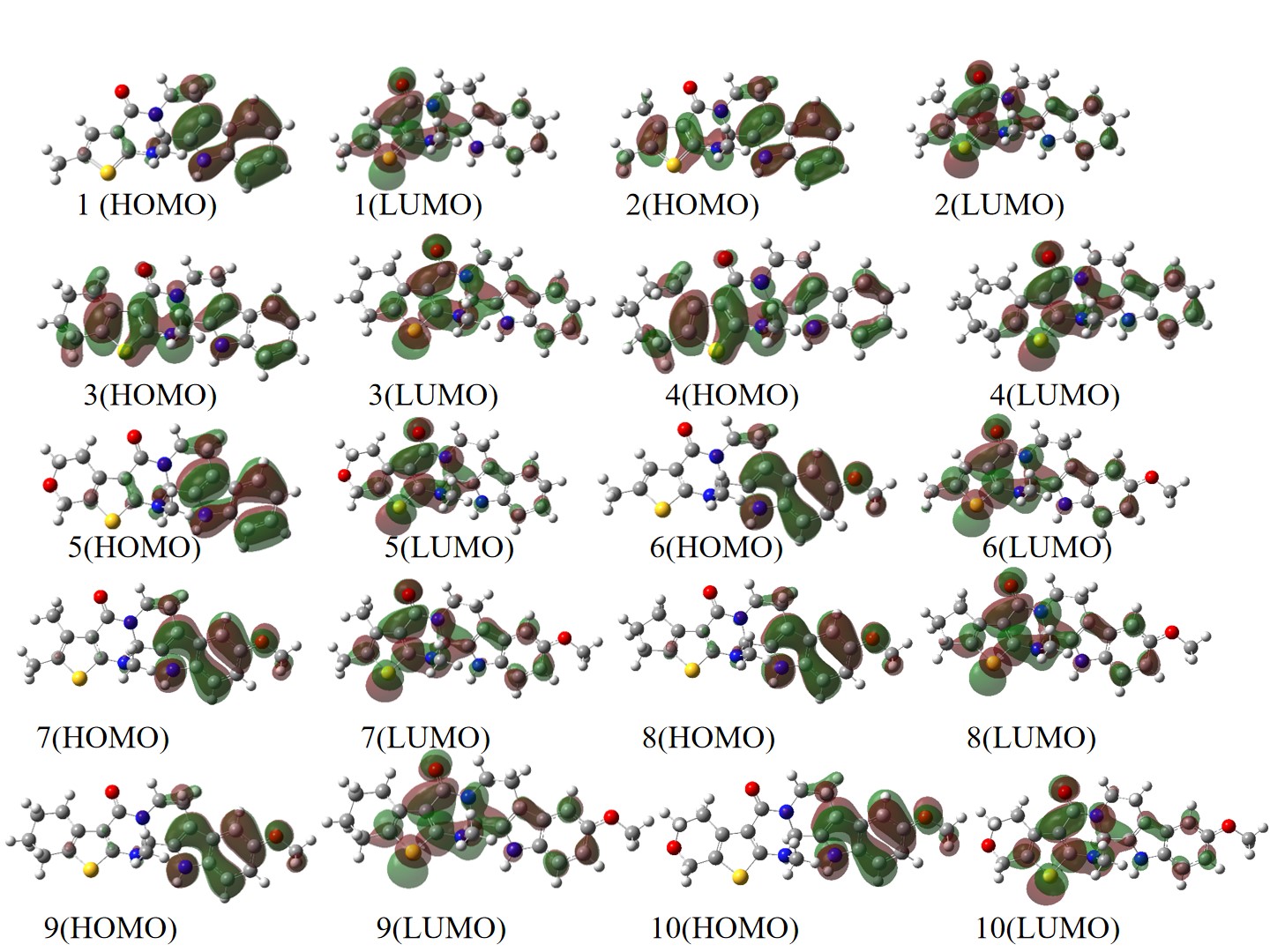


**Figure S3**. Views on the electronic iso-surfaces of the HOMO and LUMO of the optimized structures of the ten derivatives obtained by using the B3LYP/6-311++G(d,p) method


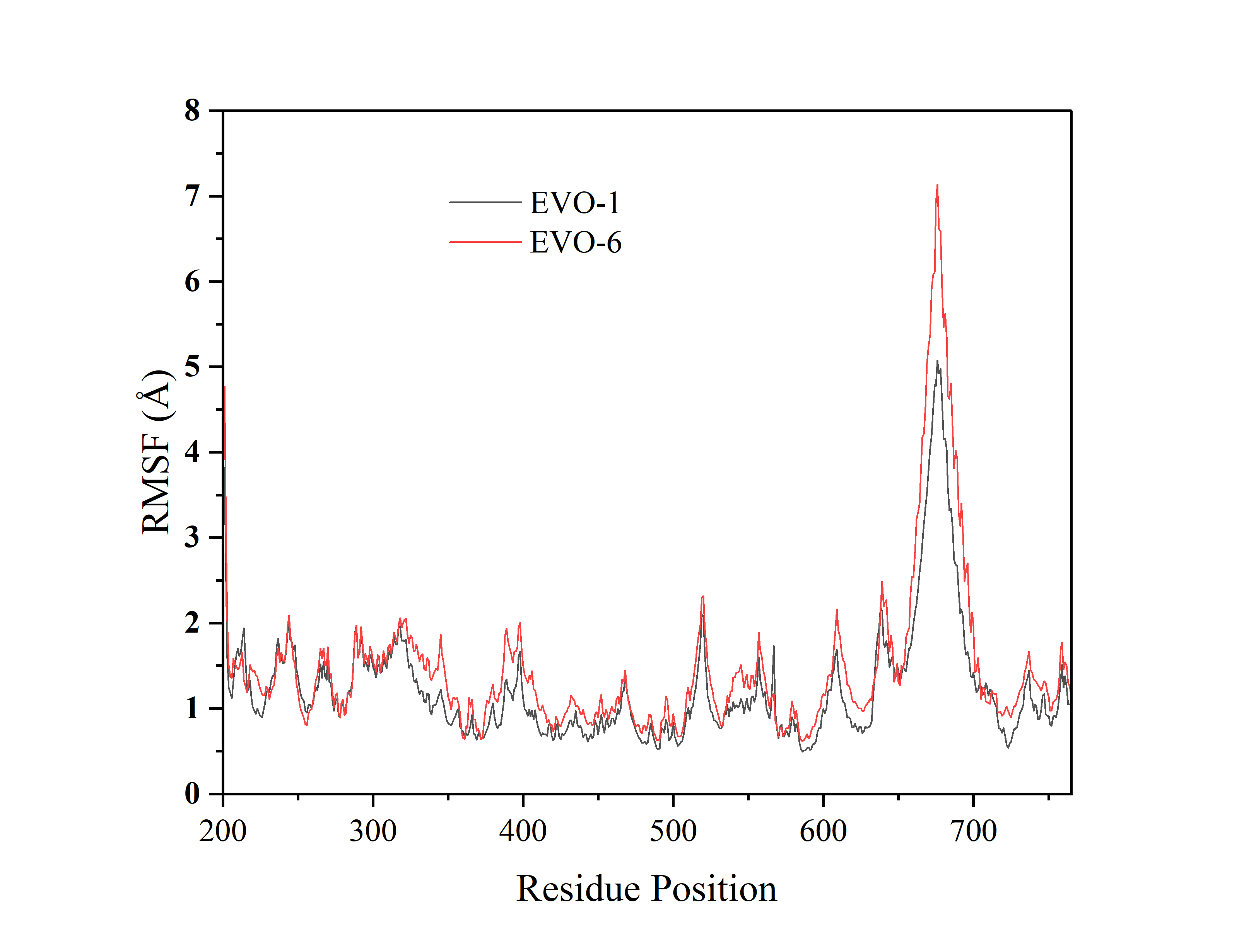


**Figure S4.** RMSF variations of the C_α_ atom of EVO-1-TOP1, and EVO-6-TOP1 complexed systems from MD simulation.


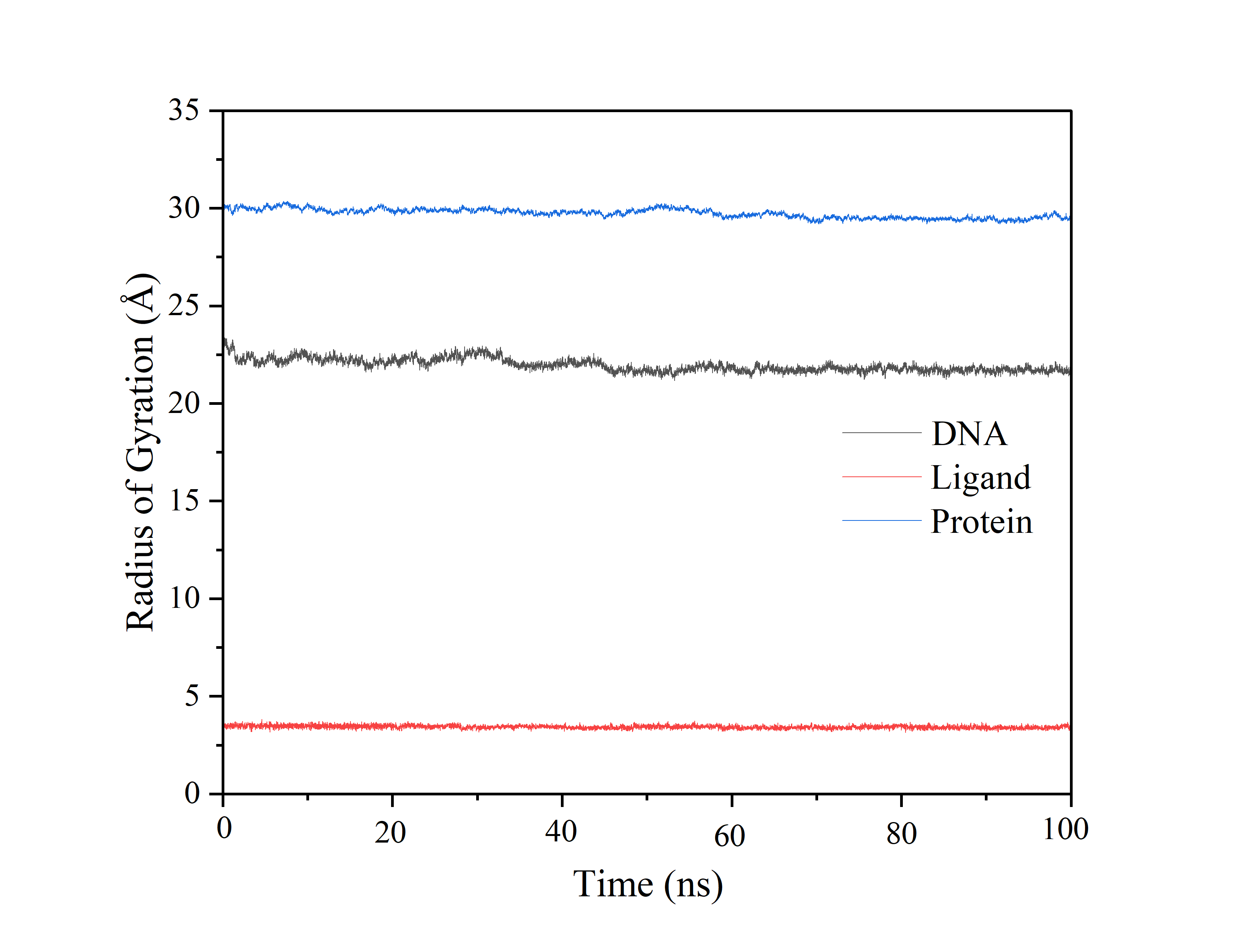


**Figure S5.** The radius of gyration of DNA, ligand and protein with 100 ns MD simulations for the TOP1/EVO-1 system.


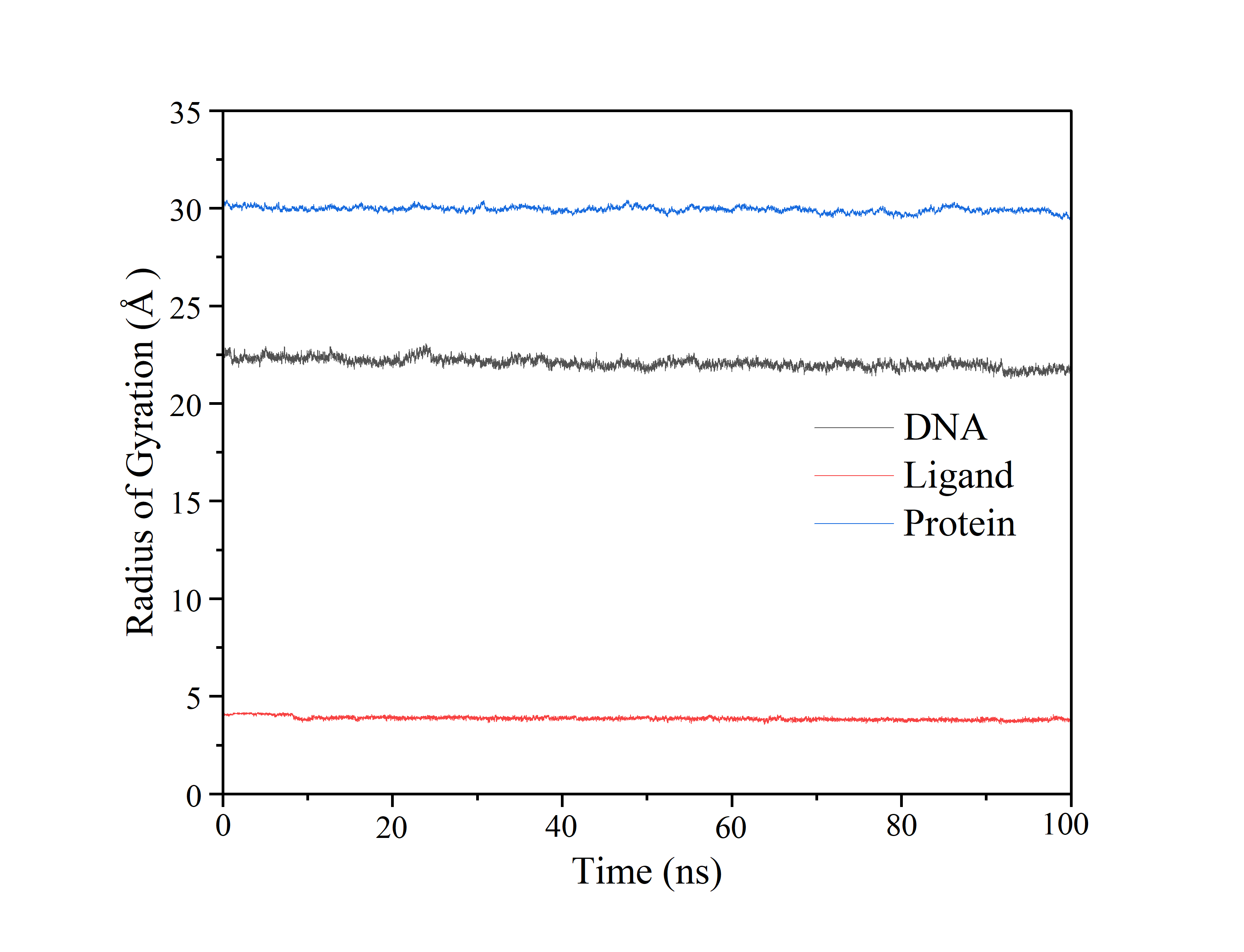


**Figure S6.** The radius of gyration of protein, DNA, and ligand with 100 ns MD simulations for the TOP1/EVO-6 system.


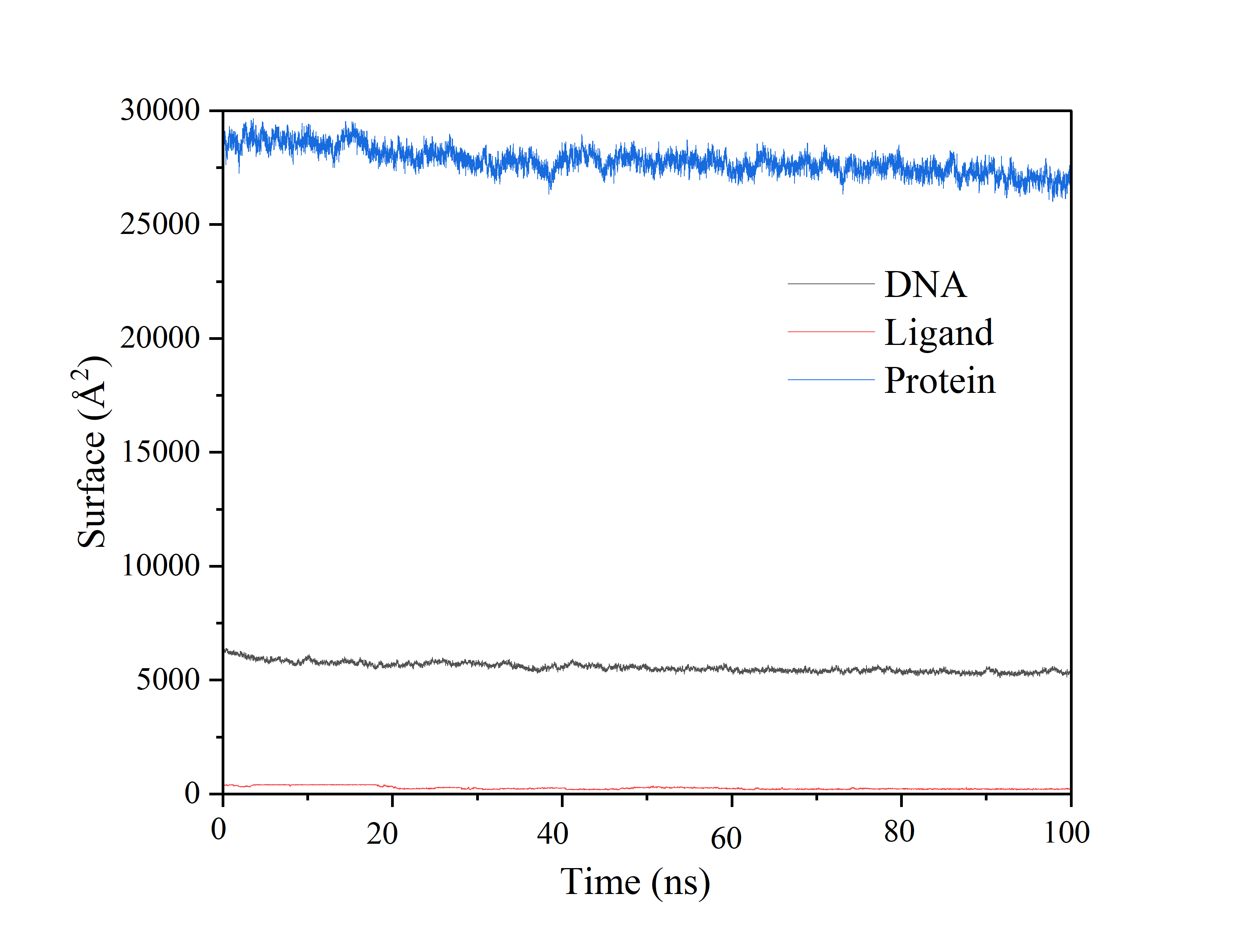


**Figure S7.** The surface of DNA, ligand and protein with 100 ns MD simulations for the TOP1/EVO-1 system.


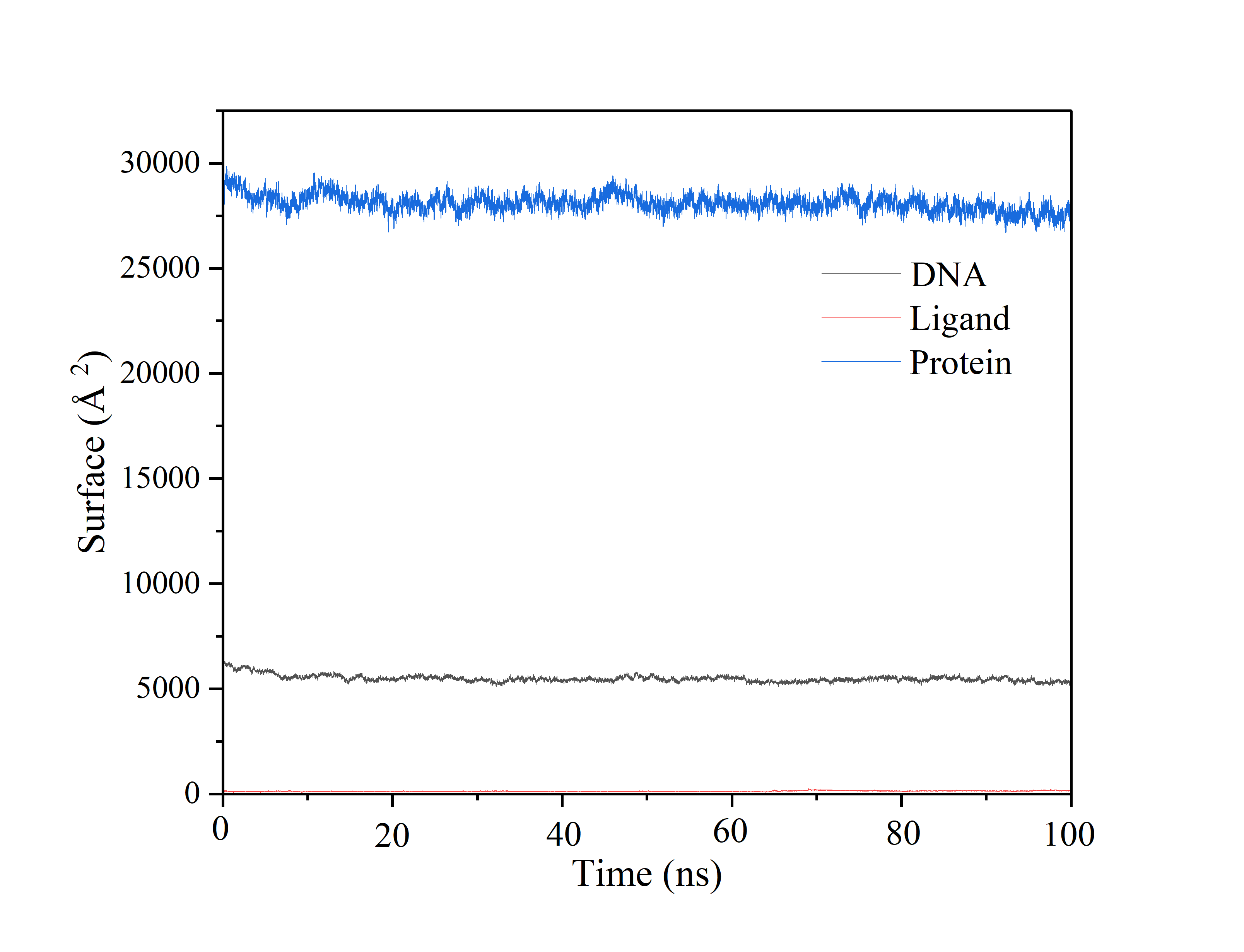


**Figure S8.** The surface of DNA, ligand and protein with 100 ns MD simulations for the TOP1/EVO-6 system.


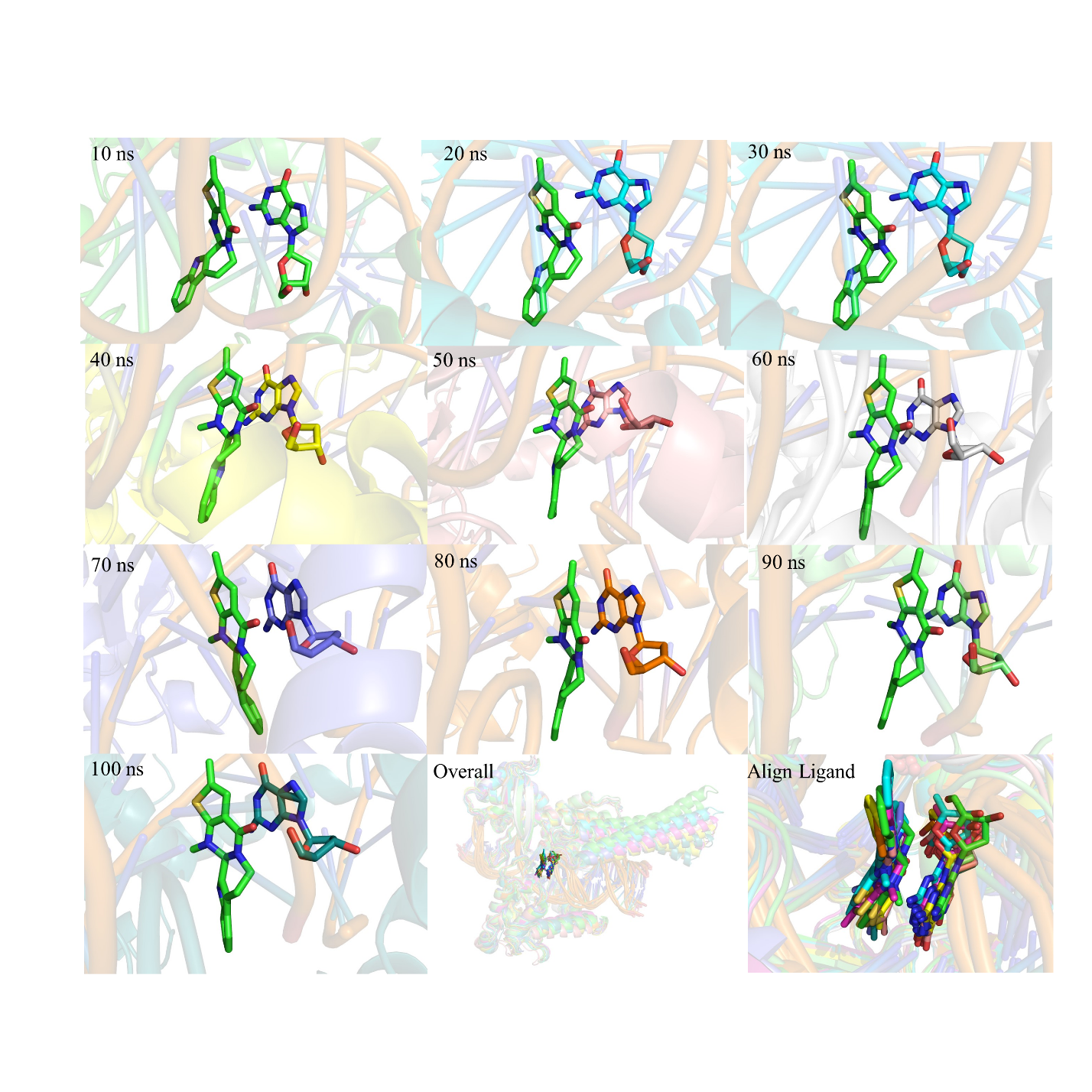


**Figure S9.** Snapshots of the TOP1/EVO-1 system along the dynamic simulation time for 10, 20, 30, 40, 50, 60, 70, 80, 90, and 100 ns and also their aligned form. For clarity, the water molecules have been removed. The inhibitor is plotted using the stick style (green for EVO-1), while TOP1 is plotted using the cartoon style.


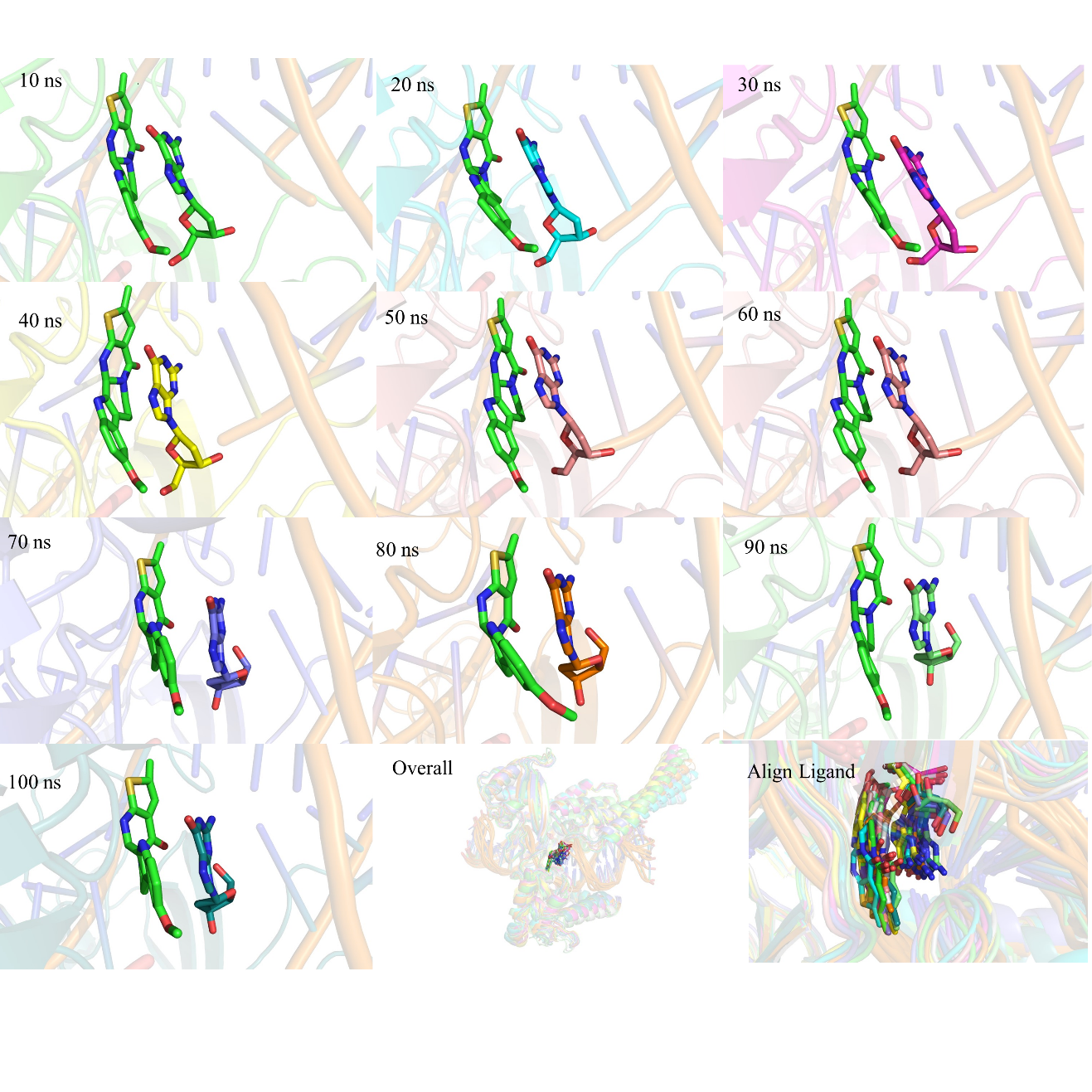


**Figure S10.** Snapshots of the TOP1/EVO-6 system along the dynamic simulation time for 10, 20, 30, 40, 50, 60, 70, 80, 90, and 100 ns and also their aligned form. For clarity, the water molecules have been removed. The inhibitor is plotted using the stick style (green for EVO-6), while TOP1 is plotted using the cartoon style.

**Table S1.** Binding free energy of compound EVO-1 binding with TOP1 protein complexes and decomposition to electrostatic interaction, van der Waals interaction, solvation free energies, and entropy.

| Energy (kcal/mol) | Complex | | Receptor | | Ligand | | Delta | |
| --- | --- | --- | --- | --- | --- | --- | --- | --- |
|  | Average | Std. Dev.^*^ | Average | Std. Dev. | Average | Std. Dev. | Average | Std. Dev. |
| $E_{\mathrm{vdW}}$ | -5683.65 | 32.61 | -5631.57 | 32.60 | -4.00 | 0.76 | -48.07 | 2.09 |
| $E_{\mathrm{ele}}$ | -44501.09 | 240.51 | -44447.80 | 240.18 | -43.33 | 1.02 | -9.96 | 2.72 |
| $E_{\mathrm{GB}}$ | -12794.89 | 221.48 | -12801.56 | 221.47 | -15.17 | 0.35 | 21.84 | 2.14 |
| $E_{\mathrm{surf}}$ | 239.72 | 1.80 | 239.96 | 1.79 | 2.82 | 0.01 | -3.07 | 0.14 |
| $G_{\mathrm{gas}}$ | -12701.12 | 240.21 | -12706.20 | 240.20 | 62.18 | 4.13 | -57.10 | 2.77 |
| $G_{\mathrm{solv}}$ | -12555.17 | 220.92 | -12561.59 | 220.87 | -12.36 | 0.35 | 18.78 | 2.12 |
| $G_{\mathrm{gas}}+G_{\mathrm{sol}}$ | -25256.30 | 76.72 | -25267.80 | 76.76 | 49.82 | 4.17 | -38.33 | 2.24 |

* The uncertainties for all of terms are included in the parentheses, which were calculated as the root mean square error for all of frames extracted in the MM/GBSA running.

$E_{\mathrm{vdW}}$: contribution to the free energy of binding from van der Waals energy;

$E_{\mathrm{ele}}$: contribution to the free energy of binding from electrostatic energy;

$E_{\mathrm{GB}}$: contribution to the free energy of binding from polar solvation energies;

$E_{\mathrm{surf}}$: contribution to the free energy of binding from nonpolar solvation energies;

$G_{\mathrm{gas}}$: contribution to the free energy of binding from $E_{\mathrm{vdW}}$ + $E_{\mathrm{ele}}$;

$G_{\mathrm{solv}}$: contribution to the free energy of binding from $E_{\mathrm{GB}}$ + $E_{\mathrm{surf}}$.

**Table S2.** Binding free energy of compound EVO-6 binding with TOP1 protein complexes and decomposition to electrostatic interaction, van der Waals interaction, solvation free energies, and entropy.

| Energy (kcal/mol) | Complex | | Receptor | | Ligand | | Delta | |
| --- | --- | --- | --- | --- | --- | --- | --- | --- |
|  | Average | Std. Dev.^*^ | Average | Std. Dev. | Average | Std. Dev. | Average | Std. Dev. |
| $E_{\mathrm{vdW}}$ | -5711.63 | 34.24 | -5654.18 | 34.08 | -4.45 | 0.89 | -53.01 | 1.86 |
| $E_{\mathrm{ele}}$ | -44294.94 | 170.49 | -44215.98 | 170.62 | -81.58 | 1.29 | 2.62 | 2.66 |
| $E_{\mathrm{GB}}$ | -13037.19 | 150.94 | -13040.95 | 150.75 | -16.40 | 0.45 | 20.16 | 2.33 |
| $E_{\mathrm{surf}}$ | 238.46 | 2.34 | 239.27 | 2.32 | 3.12 | 0.01 | -3.93 | 0.15 |
| $G_{\mathrm{gas}}$ | -12477.36 | 178.28 | -12505.32 | 178.14 | 77.45 | 4.50 | -49.48 | 2.95 |
| $G_{\mathrm{solv}}$ | -12798.73 | 150.01 | -12801.68 | 149.81 | -13.27 | 0.44 | 16.23 | 2.32 |
| $G_{\mathrm{gas}}+G_{\mathrm{sol}}$ | -25276.08 | 82.75 | -25307.00 | 82.37 | 64.17 | 4.52 | -33.25 | 2.11 |

* The uncertainties for all of terms are included in the parentheses, which were calculated as the root mean square error for all of frames extracted in the MM/GBSA running.

$E_{\mathrm{vdW}}$: contribution to the free energy of binding from van der Waals energy;

$E_{\mathrm{ele}}$: contribution to the free energy of binding from electrostatic energy;

$E_{\mathrm{GB}}$: contribution to the free energy of binding from polar solvation energies;

$E_{\mathrm{surf}}$: contribution to the free energy of binding from nonpolar solvation energies;

$G_{\mathrm{gas}}$: contribution to the free energy of binding from $E_{\mathrm{vdW}}$ + $E_{\mathrm{ele}}$;

$G_{\mathrm{solv}}$: contribution to the free energy of binding from $E_{\mathrm{GB}}$ + $E_{\mathrm{surf}}$.

**Table S3.** Free energy decomposition of the TOP1/EVO-1 complex system at the level of individual residues into contributions from van der Waals energy, electrostatic interaction energy, nonpolar solvation free energy, polar solvation free energy, backbone energy, and side chain energy.

| Residue | $\Delta E_{vdW}$ | $\Delta E_{ele}$ | $\Delta G_{sol,GB}$ | $\Delta G_{sol,np}$ | $\Delta G_{subtotal}$ | $S\Delta G_{subtotal}$ | $B\Delta G_{subtotal}$ |
| --- | --- | --- | --- | --- | --- | --- | --- |
| Protein R364 | -2.87 | -1.03 | 0.85 | -0.38 | -3.43 | -3.41 | -0.02 |
| DNA DT 9 | -0.22 | 0.74 | -0.78 | 0.00 | -0.22 | -0.28 | 0.06 |
| DNA DT 10 | -2.48 | -0.28 | 0.48 | -0.14 | -2.41 | -2.31 | -0.10 |
| DNA TGP 11 | -5.68 | -2.72 | 0.27 | -0.46 | -8.59 | -8.59 | 0.00 |
| DNA DG 12 | -0.88 | 0.81 | -0.03 | -0.07 | -0.15 | -0.82 | 0.68 |
| DNA DC 112 | -2.37 | -1.81 | 3.75 | -0.24 | -0.65 | -0.71 | 0.06 |
| DNA DA 113 | -5.21 | -0.12 | 1.73 | -0.40 | -3.96 | -3.64 | -0.33 |

Energies are in kcal/mol.

$\Delta E_{vdW}$: contributions from van der Waals energy;

$\Delta E_{ele}$: contributions from electrostatic interaction energy;

$\Delta G_{sol,GB}$: contributions from polar solvation free energy;

$\Delta G_{sol,np}$: contributions from nonpolar solvation free energy;

$\Delta G_{subtotal}$: contributions from binding free energy;

$S\Delta G_{subtotal}$: contributions from side chain energy;

$B\Delta G_{subtotal}$: contributions from backbone energy.

**Table S4.** Free energy decomposition for the TOP1/EVO-6 complex system at the level of individual residues into contributions from van der Waals energy, electrostatic interaction energy, nonpolar solvation free energy, polar solvation free energy, backbone energy, and side chain energy.

| Residue | $\Delta E_{vdW}$ | $\Delta E_{ele}$ | $\Delta G_{sol,GB}$ | | $\Delta G_{sol,np}$ | $\Delta G_{subtotal}$ | $S\Delta G_{subtotal}$ | $B\Delta G_{subtotal}$ |
| --- | --- | --- | --- | --- | --- | --- | --- | --- |
| Protein A351 | -0.89 | -0.39 | | 0.82 | -0.21 | -0.67 | -0.60 | -0.07 |
| Protein R364 | -0.52 | -2.50 | | 2.20 | -0.02 | -0.84 | -0.81 | -0.03 |
| Protein P431 | -0.70 | -0.15 | | 0.31 | -0.18 | -0.73 | -0.71 | -0.02 |
| DNA DT 10 | -3.10 | -0.19 | | 1.02 | -0.32 | -2.57 | -2.38 | -0.19 |
| DNA TGP 11 | -6.72 | 2.13 | | 0.84 | -0.60 | -4.35 | -4.35 | 0.00 |
| DNA DC 112 | -2.72 | 1.08 | | 0.32 | -0.17 | -1.47 | -1.18 | -0.30 |
| DNA DA 113 | -5.24 | 0.15 | | 1.10 | -0.35 | -4.31 | -3.71 | -0.60 |

Energies are in kcal/mol.

$\Delta E_{vdW}$: contributions from van der Waals energy;

$\Delta E_{ele}$: contributions from electrostatic interaction energy;

$\Delta G_{sol,GB}$: contributions from polar solvation free energy;

$\Delta G_{sol,np}$: contributions from nonpolar solvation free energy;

$\Delta G_{subtotal}$: contributions from binding free energy;

$S\Delta G_{subtotal}$: contributions from side chain energy;

$B\Delta G_{subtotal}$: contributions from backbone energy.
